# Institutional Courage in Healthcare: An Improvement Project Exploring the Perspectives of Veterans Exposed to Airborne Hazards

**DOI:** 10.3390/bs13050423

**Published:** 2023-05-17

**Authors:** Katharine Bloeser, Mikayla McAdams, Kelly K. McCarron, Samantha Varon, Lisa Pickett, Iman Johnson

**Affiliations:** 1 The War Related Illness and Injury Study Center, The VA New Jersey Health Care System, 285 Tremont Ave., East Orange, NJ 07019, USA; 2 Silberman School of Social Work at Hunter College, The City University of New York, New York, NY 10035, USA; 3 VA Providence Health Care System, Providence, RI 02908, USA; 4 School of Public Health and Tropical Medicine, Tulane University, New Orleans, LA 70112, USA

**Keywords:** institutional courage, institutional betrayal, airborne hazards, veterans, healthcare management, healthcare policy

## Abstract

Background: Military environmental exposures and care for subsequent health concerns have been associated with institutional betrayal, or a perception on the part of veterans that the US government has failed to adequately prevent, acknowledge, and treat these conditions and in doing so has betrayed its promise to veterans. Institutional courage is a term developed to describe organizations that proactively protect and care for their members. While institutional courage may be useful in mitigating institutional betrayal, there is a lack of definitions of institutional courage in healthcare from the patient perspective. Methods: Using qualitative methods, we sought to explore the notions of institutional betrayal and institutional courage among veterans exposed to airborne hazards (i.e., airborne particulate matter such as open burn pits; N = 13) to inform and improve clinical practice. We performed initial interviews and follow-up interviews with veterans. Results: Veterans’ depictions of courageous institutions contained key themes of being accountable, proactive, and mindful of unique experiences, supporting advocacy, addressing stigma related to public benefits, and offering safety. Veterans described institutional courage as including both individual-level traits and systems or organizational-level characteristics. Conclusions: Several existing VA initiatives already address many themes identified in describing courageous institutions (e.g., accountability and advocacy). Other themes, especially views of public benefits and being proactive, hold particular value for building trauma-informed healthcare.

## 1. Introduction

Institutional betrayal, or the failure of institutions to protect or care for its members [1], has been observed in military and healthcare experiences among military veterans [2,3,4,5]. One type of stressor that has been associated with institutional betrayal is environmental exposures (i.e., exposure to potentially hazardous toxins) during military service. Environmental exposures are a wide-reaching problem in the veteran community; 37% of veterans seeking care in the Department of Veterans Affairs (VA) healthcare system report concerns about environmental exposures [6]. While there is increasing research on the importance of addressing institutional betrayal in healthcare, there is little empirical data on the factors that can protect against institutional betrayal. The existing literature has proffered institutional courage as a way for organizations to proactively prevent institutional betrayal and respond to crises or trauma in a manner that supports rather than admonishes victims [7,8]. The current study seeks to identify a definition of institutional courage in healthcare within a cohort of veterans with a history of environmental exposures who reported that they have experienced institutional betrayal. This definition, especially from veterans’ perspectives, is crucial for improving care for veterans with environmental exposure concerns and other occupational stressors in which institutional betrayal may be common. 

Environmental exposures have been known to occur in spaces where military service members live and work and have been associated with perceptions of institutional betrayal [2]. For example, in the 1980s, many veterans and their families living at Camp Lejeune were exposed to dry cleaning chemicals associated with cancers and other chronic diseases through contaminated drinking water [9,10]. Foams developed for firefighting and used on military bases exposed military families and surrounding communities to substances that are now known to be associated with adverse reproductive and immunological effects [11]. Historically, combat eras have been marked by prevalent environmental exposure concerns. It is now known that Vietnam veterans exposed to Agent Orange, an herbicide used throughout the combat theater, are at greater risk for developing cancers, ischemic heart disease, stroke, hypertension, and type 2 diabetes mellitus [12]. The Gulf War illness (GWI) is a medical condition associated with environmental exposures during the 1991–1992 Persian Gulf War [13] and is characterized by fatigue, chronic wide-spread pain, cognitive difficulties, changes in mood, and gastrointestinal problems [14,15]. 

While deployed to the Persian Gulf region in both 1991–1992 and in the decades after September 2001, many US and allied military members experienced exposure to airborne hazards, including pollution (e.g., smoke from open burn pits of refuse and trash), fine particulate matter (e.g., sand and dust), and fumes [16]. Airborne hazard exposures are associated with a variety of health conditions, including cancers and respiratory diseases [16,17]. These exposures have become the hallmark military exposure for veterans who served in Afghanistan and Iraq during Operation Enduring Freedom (OEF) and Operation Iraqi Freedom (OIF). Research suggests that veterans exposed to airborne hazards may feel betrayed by the US government and healthcare system as they face uncertainty about symptoms associated with airborne hazard exposures and care for those conditions [18]. The PACT Act, which was supported by advocacy from veterans, was signed into law in 2022 and directs VA to improve care for veterans with airborne hazard exposures [19].

Previous work on institutional betrayal among veterans have focused on two military stressors, GWI and military sexual trauma (MST). Research documents veterans’ belief that the Department of Defense (DoD) and VA have systematically denied the existence of GWI and its connection with environmental exposures [2,20]. This perceived institutional betrayal has extended into the healthcare offered to veterans for their GWI, whereby veterans feel providers obfuscate the cause of GWI and its treatment [2]. For veterans who experienced MST (i.e., sexual harassment and/or sexual assault while in the military), institutional betrayal has occurred in both the failure of the DoD to prevent MST from occurring, as well as the subsequent victimization of those who report MST [3,21]. Institutional betrayal among veterans who experienced MST is associated with depression, trauma-related symptoms [22], suicide attempts [4], disillusionment with the institution of the military, and not wanting to use VA healthcare [5]. Some survivors of MST feel that the ensuing institutional responses to MST were more harmful than the experience of the assault itself [22,23]. As such, in addition to more well-recognized military stressors, institutional betrayal is central to veterans’ experience with healthcare [24,25]. 

Descriptions of institutional courage can direct improvements in care for veterans who have environmental exposure concerns as well as other military-based experiences of institutional betrayal (e.g., MST) [2,21,22,23]. To date, there has been little empirical investigation into the definitions of institutional courage and its operationalization in everyday practice. The existing literature suggests that both the institutions themselves and the individuals within institutions (through contributions, action/inaction) can demonstrate institutional courage [26]. Courageous institutions are described as transparent, protective [1], accountable, and active in seeking justice for victims [26]. Courageous institutions also examine how they perpetuate institutional betrayal, provide effective means for members to report problems, adjust to the needs of members who experienced harm, and provide reparations to victims [1]. After presenting a brief description of veterans’ experiences of institutional betrayal related to healthcare for environmental exposures, we will add to the literature on institutional courage by further elucidating a definition of institutional courage in healthcare from the patient perspective. In doing so, we will also help identify the organizational characteristics needed to support individuals in creating courageous institutions in healthcare. We also hope to inform future efforts aimed at fostering institutional courage in clinical practice with this population of veterans as directed by the PACT Act. 

## 2. Materials and Methods

### 2.1. Context

The Veterans Health Administration (VHA) provides healthcare to eligible veterans of the US military. Eligibility for VA healthcare is dependent upon a variety of factors, including financial means, disability, and era of service [27]. The healthcare benefits which veterans receive can be dependent upon the receipt of VA compensation and pension benefits, which are benefits considered to be connected to military service and are often referred to as service-connection. For example, a veteran must meet certain disability levels (i.e., percentage) to qualify for long-term care, including nursing home care [27]. This compensation and pension process is governed by the Veterans Benefits Administration (VBA) [27]. Service connection is a cash benefit given to veterans with disabilities that resulted from or were made worse by military service; Congress approves conditions that qualify for disability benefits [28]. The approval of such conditions has been the focus of advocacy on the part of veterans and their families, including conditions associated with Agent Orange exposure during the Vietnam War [29] and exposures during the wars in the Persian Gulf region in 1991–1992 [20]. The War-Related Illness and Injury Study Centers (WRIISCs) are tertiary referral centers designed to provide clinical consultation, research, and education on post-deployment health conditions, including airborne hazard concerns [30]. In 2017, researchers and clinicians at the WRIISC developed a quality improvement project to evaluate the experiences of institutional betrayal and institutional courage among veterans seen at the WRIISC to inform and improve future WRIISC education efforts and research. This project was designated quality improvement by the VA New Jersey Health Care System’s Institutional Review Board (IRB).

### 2.2. Participants

Participants were seen clinically at the WRIISC for a comprehensive evaluation of their post-deployment health concerns, including airborne hazard exposures in the previous three years. If a project team member participated in the veteran’s clinical evaluation, they did not interview the veteran. We approached 22 veterans for interviews, expecting half to decline given the length and depth of the interviews. While no veterans explicitly declined to be interviewed, 9 did not respond to invitations to participate. Participants included veterans who identified as men (*n* = 8) and women (*n* = 5). All veterans were deployed to the Persian Gulf Region. Participants were approached by a non-clinical team member, who was previously not known to the veteran. 

### 2.3. Data Collection

The present work focuses on interviews conducted with veterans exposed to airborne hazards. Two interviews were conducted; the first interview lasted between 45 min and 5 h and used a phenomenological lens [31] to examine veterans’ experiences with the notions of institutional betrayal and institutional courage. Of note, longer interviews were broken into two-hour increments to avoid fatigue. This interview used a semi-structured interview guide that provided a definition of institutional betrayal and institutional courage and then asked about the veterans’ experiences with these two phenomena generally, in VA healthcare, and as they related to exposure to airborne hazards. This interview was transcribed verbatim by the VA’s Centralized Transcription Services Program. The second interview was more conversational in tone and sought to position the team and veteran participants as collaborators in developing project outcomes (e.g., patient handouts and education materials for providers) [32,33]. This interview asked veterans about how to best address institutional betrayal and institutional courage in VA healthcare. Notes were taken throughout the second interview, including documenting veterans’ words verbatim when appropriate. While the IRB deemed this work quality improvement, participants signed a consent-to-record form and were informed of the use of data. 

### 2.4. Data Analysis

The team engaged in debriefing sessions after the first veteran interview. Debriefing sessions followed a pre-specified format to both support the interviewer, inform the interview guide, and formalize analytic memo writing to develop themes across interviews. The team followed the Birks et al. [34] procedure for analytic memo writing whereby the team documented decision-making for the entire process (e.g., future interviews, theme saturation, and project outcomes), extracted meaning from the data (e.g., initial open coding, themes to explore in the second interview), discussed the team’s perspectives and reflexivity over the course of the project, and offered open communication among team members. 

Upon completion of the first round of interviews, the team started an iterative process to develop a codebook. Team members, in dyads, first read through the same transcript and developed an initial list of open codes. The dyad then met to collaborate on a list of codes for the shared transcript. This list was then shared with the whole team who then discussed the inclusion of the code in the initial codebook and its definition. Once the team had evaluated three transcripts in dyads, these same dyads coded the remaining transcripts and met to reach a consensus on the application of the codebook. Discordance between coders was resolved using a meeting with the team at large. Throughout this process, the team continued to meet regularly to further refine codes and definitions, add to the initial codebook, and discuss and formalize thematic codes. NVivo was used to organize data analysis. We were also informed by the Standards for Quality Improvement Reporting Excellence (SQUIRE 2.0) Guidelines [35]. 

## 3. Results

We first briefly summarized themes related to the veterans’ experiences with institutional betrayal, both in the military and in healthcare, to provide perspective and context for the veterans’ discussion of institutional courage. We then looked to the veteran interviews for themes related to a definition of institutional courage and characteristics of courageous institutions. 

### 3.1. Experiences of Institutional Betrayal

In this sample of veterans living with complex health conditions related to airborne hazards and other environmental exposures, the veterans described instances of institutional betrayal both within and outside of military service. In terms of environmental exposures while in the military, the veterans described feeling that they were either not warned about exposures or given proper protective equipment. Veterans also described retaliation when reporting wrongdoing or dangerous situations. This was the experience of a veteran who reported safety concerns in their workplace:

*Absolutely nothing was done and after I filed a…complaint, retaliation ensued and the commander…told me that if I didn’t stop turning in safety violations that I would lose my job. And so I ended up losing my job*.

Participant 2.

Veterans often felt as though they were lied to by the military about other exposures. For example, the government denied that they were exposed to substances they believe they were in contact with or that the government downplayed its severity. 

Veterans also described other experiences related to their overall military service and when seeking healthcare. For instance, one veteran described being discharged from the military under Don’t Ask Don’t Tell (DADT), the policy that did not allow open service for sexual minority service members (see Burks 2011 [36] or Kerrigan 2012 [37]). Here, the cruelty and injustice of DADT extended to the actions of several individuals in the military who enacted the policy. In healthcare settings, veterans felt that they were not believed when describing the severity of their symptoms, their providers were more focused on “policing” (Participant 9) or had a need to “be right” rather than “empathetic” (Participant 10), and that the US government did not keep their promise to care for their military-related health conditions. As Participant 3 states, “essentially the system lied to me.” The idea of policing seemed to overlap with labeling. For multiple veterans, they felt that if they were pleasant with providers they were not taken seriously, yet if they were more assertive, they would be labeled as hostile. A veteran described receiving negative results from testing for certain conditions and being labeled as malingering. 

It should also be noted that veterans described experiencing institutional betrayal from society at large, beyond the military, VA, and the health care system. For several veterans, this was in the form of discrimination as veterans and/or as people of color in their civilian jobs. This came in the form of verbal harassment and physical harm while at work. 

### 3.2. Defining Institutional Courage

While the term institutional courage resonated with veterans, it was challenging to draw a discrete definition of institutional courage from the data. One veteran described institutional courage as being akin to the army core values “and the L.D.R.S.H.I.P. acronym: loyalty, duty, respect, selfless service, honor, integrity, personal courage” (Participant 8). Another veteran was able to describe a lack of institutional courage when the military allowed unsafe conditions to continue, “they could have pulled the plug on things in some of those situations a lot sooner than they did” (Participant 12). One challenge in developing a definition of institutional courage from the data was separating out and drawing on individual acts of courage that occurred while veterans were in the military. These acts were committed by the veterans themselves and those they served with and were shaped by the critical nature of their missions. For example, one veteran described serving as a medical provider who triaged and treated hundreds of combat casualties. Other veterans described incidents in which they placed themselves in danger to protect the lives of others in their unit. Veterans’ view of institutional and/or individual courage in civilian life seems to be informed in relation to their experiences of courage in wartime.

### 3.3. Characteristics of Courageous Institutions 

What emerged readily from the data were characteristics of courageous institutions. Veterans spoke about VA, VHA, and VBA as institutions and spoke to institutional courage in healthcare generally. Veterans described six characteristics of courageous institutions: being accountable, proactive, and mindful of unique experiences, valuing advocacy, supporting a cultural shift around social welfare, and offering safety. 

#### 3.3.1. Being Accountable

Veterans discussed the notion that courageous institutions have active and strong accountability for members. This was described in three ways. First, as a means of operation—having systems to hold individuals in the institution accountable. For one veteran, accountability involved consequences to actions, or “teeth in it,” for example, “you have X amount of days to respond” (Participant 3) to a patient request. Another veteran states that the organization should have “ethical intelligence” or “if someone wants to actually provide value to society—that’s the goal—they have to actively create something that has a process for checks and balances” (Participant 2). 

Second, accountability also seemed linked to the idea that VA should hold the government accountable for the sacrifices made by the veteran. Failure to uphold this obligation was discussed by veterans. Participant 13 felt that the VA “has ignored or denied my injuries…what I was exposed to by my own government is going to kill me, and yet the same—my own government doesn’t want to help me, either.” As Participant 8 stated, “I believe in doing what you’re told and giving your all, I did what I was expected to do. And it ended up causing me to be injured.” Veterans raised the idea that VA was a space for reparations from the past, particularly betrayal that occurred within the military. As this veteran states, the VA was the only means of recourse or repair from conditions that originated during their time in the military:

*I think because we’re never going to get the justice that we were looking for from our branch of service. Matter of fact, you know, anything but it. You know as soon as you get your discharge, you’re done, goodbye…it’s like they don’t want to hear from you. So all we have left is the VA*.

Participant 1.

Another veteran described holding VA accountable for their role in repairing injuries from the military, stating that they had hoped the VA “would assist me more, would’ve actually fought for me more” (Participant 12). Similarly, participants described feeling that if the VA does not help veterans there is little help outside VA. 

Third, veterans saw themselves as part of this process of accountability, perhaps stepping in to fill gaps in VA and American society. Veterans saw themselves as accountable to each other and thus were an intricate part of supporting VA as an institution. This meant helping veterans navigate the system, “I do believe that all this has happened to me for a reason. So I actually see what I go through as a gift… And maybe I was meant to go through this so some other veterans won’t… So I don’t see it as a curse, I see it as a blessing” (Participant 13). This also meant reaching out to other veterans to “get my story heard, two, get other veterans to hear it and want to sign up for the burn pit registry” (Participant 17). 

#### 3.3.2. Proactive

Veterans shared a desire for VHA to be more proactive and “preventative” (Participant 7 and Participant 13) in their efforts to treat veterans for military exposure concerns. This was especially important to veterans who felt that their condition was “progressive” (Participant 9) or that their prognosis was poor. Another veteran described the feeling that had their care been more proactive when their symptoms first started, “I wouldn’t be in the pain that I’m in now” (Participant 11). One veteran noted that they wanted their provider to be “really investigating…taking the time and…[asking] what might have caused this?” in reference to their medical conditions (Participant 1). Another veteran stated, “…if you don’t do the courageous thing of standing up and being preventative…they give you monetary compensation once you’re so sick that you’re in bed all the time” (Participant 7). Similarly, “I think it would behoove the VA to be…a little bit more proactive and do workups on people instead of waiting until… people are unable to do things” (Participant 7). This included assigning veterans to primary care providers based on the “health needs of the vet” and not on the doctor’s availability (Participant 13) and specific outreach to newer cohorts of veterans:

*…if there was a survey that asks you, do you have this symptom, this symptom, this symptom, this symptom?…if you scored over a certain point on that system I think you should automatically be sent for certain testing*.

Participant 20.

One Veteran stated, “I didn’t know that I was even eligible to get medical care…after I exited the service” (Participant 11) suggesting it was the VA’s responsibility to reach out and inform them of the benefits they had earned. Another veteran described a desire for VA to note lapses in care and “zero contact” from VA (Participant 1). This participant described that they had been seeing a provider who left the VA; the veteran went on to receive care outside VA but was never contacted to ask about their welfare or need for a new provider. This veteran similarly defined being proactive as “they would put more time into checking the guys” who had been exposed to combat trauma (Participant 6). Another veteran offered a concrete solution:

*I think every single one of us should have a caseworker, or a social worker who is calling us every 90 days. Whether you’re a healthy vet or not…somebody should be calling me every 90 days going, are you making it to your doctor’s appointments? Are you getting the doctor’s appointments you need? Do you have a safe place to live? Do you have food on your table?*

—Participant 12.

#### 3.3.3. Mindful of Unique Experiences

Many veterans described the feeling that a one-size-fits-all approach to their care was a form of betrayal. For these veterans, they wanted a system that adapted to their unique needs and “accept people where they’re at and assist them to thrive where they’re at” (Participant 7). As this veteran states:

*Because they don’t fit into their system, they’re not willing to work with them individually to be able to learn how to fit into the system…it’s the same way with the VA, it’s the same way with schools, it’s the same way with other places…I don’t think that a lot of places want to take the, have the courage to expand and help people to thrive in that environment*.

Participant 17.

Another veteran went on to describe a positive experience with their care, where they were treated as an individual:

*I’m not just rushed in and rushed back out. I know I’m one of those trouble cases, you know, a difficult case they call me. I get that. And so sometimes doctors are like, yeah, we’ve heard it all, duh, duh, duh, duh, here’s what you need to do, bye. And the ones that are like, okay, tell me all of it, what more can I do? I think those are the ones that do have that institutional courage*.

Participant 12.

Valuing the unique experiences of patients included a concrete recommendation from Participant 3, who noted that they wanted healthcare providers to read the entirety of their patient record or medical record and perform thorough history and physicals. This veteran stated that providers are not afforded enough time to adequately read records and effectively assess patients. The veteran then expanded on the idea that institutional betrayal often occurs in healthcare because of inadequate resources noting that providers are “overwhelmed by the numbers.” 

#### 3.3.4. Value and Support Advocacy

Veterans also discussed the value of advocacy for veterans in general and in healthcare. First, veterans expected the VA to be an advocate, “if the VA can’t be our advocate then why does the VA exist?” (Participant 1). This also applied to individual members of the institution, “I just wish more people would be willing to go outside themselves and advocate, there you go, advocate, and I mean really advocate for what needs to be done” (Participant 7). One veteran defined advocacy for oneself and others, “You have to be able to stand your ground…forcing the government to accept responsibility for what you’ve gone through” (Participant 4). Veterans also described employee advocacy as, “passion for helping us” (Participant 10) and not doing things for “recognition” (Participant 10). Here, veterans also reflected on the idea that leaders needed to advocate for their employees. This could, in some ways, offset the perils of a large bureaucracy. This veteran recalled asking their subordinates, “what do I need to do as your Commander, to get you promoted? What help do you need?” (Participant 13). For this veteran, this also meant attending to their personal lives, “if a person doesn’t have to worry about his home life, his work life is much better” (Participant 13).

As with the theme of accountability, veterans reflected on their role in VA as members of the institution. This took the form of self-advocacy. Veterans spoke of their need to engage in self-advocacy after the system let them down. As this veteran described:

*I needed to take charge of my destiny and self-advocate. And that I was not in a situation I thought I was in. I thought I was in a meritocracy, where everyone’s treated equally and fairly and that people cared about me, loved me and valued me. And none of that turned out to be true*.

Participant 8.

In one narrative, the idea of being seen as problematic or difficult can result from advocating, including when engaging in self-advocacy. 

Similar to leadership’s advocacy, for this veteran, individual advocacy on the part of employees could also compensate for difficulties inherent to large bureaucratic institutions:

*I’ll never say anything that’s universally bad or universally good…There are pockets of hope, and that makes things a little easier to deal with. You know, I get it’s a big bureaucracy, gets in its own way, but there are those who work in, within the confines of the bureaucracy that really do try to work for the benefit of the veteran*.

Participant 11.

These descriptions also included the notion of tenacity related to advocacy; that often veterans and employees had to repeatedly return to situations where they were initially turned down, discounted, or invalidated. One example was in repeatedly applying for service-connected benefits despite initial denial for compensation from VBA. Several veterans expressed a feeling of validation or relief after being approved for service-connected conditions.

#### 3.3.5. Support a Cultural Shift around Social Welfare

Veterans discussed the notion that their receipt of both medical and monetary benefits from VA at times solicited a response from providers that they were “getting a free ride” or spoken about in the following regard: “look at them getting that free money” (Participant 5). This same veteran stated, “If I could give these injuries back, and give back the money because of that, we’d do it in a heartbeat.” Another veteran described their experience:

*It is better in some parts of the country—they think they’re doing you a favor and don’t believe in entitlement—systemic prejudice in helping veterans when it comes to money—don’t even think that its conscious*.

Participant 3.

Participant 9 described feeling that they needed to prove their level of disability to their provider, perceiving the provider as saying, “we do not see anything wrong with you.” Similarly, Participant 1 said that the system should “give us the benefit of the doubt” instead of “denying” that something did or did not happen. 

Veterans also described the idea that benefits were more than merely financial compensation. As one veteran stated, “I don’t care about money” (Participant 9). This same veteran went on to say, “I’ve accomplished quite a bit in my life, and so to be labeled as disabled or anything like that is somewhat depressing and discouraging in itself.” Participant 6 also stated, “great, you service-connected me, awesome, and now I can [be] financially be stable. But now I need you to actually fix me because I can’t work because of these conditions. So, service connection is great and all, being financially [stable] is wonderful, but a long life is even better.” One veteran stated, “that compensation is not just money. That compensation for that soldier is an affirmation to his contract that he signed and earned. And that is bigger than the money” (Participant 6). Another veteran states, “I’m not asking for more money…I need the money now because I can’t work, but I just want care. I want care, empathy, understanding” (Participant 10). While the symbolic nature of benefits was noted as important, another veteran also highlighted, “I need that income to survive, for my family” (Participant 13). 

Veterans also discussed the benefits process, “I think [VBA] does most of the damage. VHA tries to do a lot of the care, but the damage is done from the benefit side” (Participant 1). Another veteran explicitly stated, “when you into the VBA side of things, you do not get the sense the VA is there to help you. You completely don’t feel that—you know, you don’t feel that the people who is evaluating you are going to try to make sure that you get the benefit” (Participant 13). At the same time, veterans spoke of VBA and VHA as being inextricably linked. Veterans shared that in VHA, they often felt that the notion of connecting their condition to military service was “taboo” and “we can’t talk about it, we can’t look at it” (Participant 4). As Participant 5 stated, “we [veterans] didn’t create the rule that’s called service connection. It was created for us.”

#### 3.3.6. Offer Safety

Participant 2 described the notion that an “ethically intelligent” organization must also recognize that there is power over individual actors within the organization; for example, employees are dependent upon the organization for their livelihood, and patients in a healthcare organization are dependent upon the organization for their care. This suggests that the organization must also recognize that individuals may not respond in courageous ways due to fear of retribution. This included that an institution made up of humans is inevitably fallible. All actors within institutions will make mistakes, as this veteran states, “I was always taught if you make mistakes you own it. That takes a lot of courage. And I think the VA, our government, they make mistakes. We’re all human. Own it” (Participant 4). Participant 11 described a situation where the provider admitting they were wrong built trust:

*[my provider] called me to tell me to stop taking that medicine right away, and if I had any further issues I needed to go to the nearest emergency room…she’s doing her job, she’s doing what’s expected of her to do. So there are two sides of that, one is that she took the initiative to call me to say, hey this could be life threatening stop taking it right away. On the other side, basically she was admitting that they are giving me a medicine that I didn’t need for a diagnosis that I did not have.*

## 4. Discussion

In our quality improvement project, veterans described instances of institutional betrayal both while serving in the military and when seeking healthcare. Veterans described the characteristics of healthcare institutions that are courageous but also, more specifically, how the VA can be a more courageous institution. First, veterans described institutionally courageous systems as having mechanisms of accountability and discussed that the VA as an institution can offer repair for betrayals that occurred during military service. Next, veterans described institutionally courageous healthcare as being proactive, both in terms of providing preventive healthcare but also proactively reaching out to veterans to ascertain their needs. Related to this was the idea that healthcare should be mindful of veterans’ unique needs and avoid a one-size-fits-all approach. Veterans also described the need for a shift in organizational culture within VA surrounding how providers view veterans’ benefits, noting that provider-level stigma surrounding public benefits has resulted in poor experiences with care. Finally, veterans described the importance of advocacy and safety in courageous healthcare systems. This includes self-advocacy, advocacy for patients, and advocacy for VA employees. Veterans described the idea that individuals should feel safe to advocate for others but also safe in admitting to errors or mistakes. 

Veterans often described individuals within institutions as being both responsible for their actions and also beholden to the rules and culture of the institution. This is consistent with the existing literature, which notes that exercising institutional courage in healthcare may be especially fraught. Interactions among healthcare organizations are complicated and interdependent; ethical clinical practice often requires personal risk when confronting structures of power [38], is inherently associated with interactions between disciplines (e.g., physicians and nurses), peers, patients, and potentially inadequate resources of the organization [39], and may rely upon the professional placing the patient’s needs before their own [40]. Thus, a conceivable problem with a focus on only the characteristics of courageous individuals within institutions is the potential for moral distress and professional burn-out within individuals [41,42] and scapegoating individuals for systemic problems within the institution [43]. The notion of institutional *and* individual responsibility for healthcare has been highlighted in the contemporary professional literature [44], especially in the wake of the COVID-19 pandemic [45]. This just culture framework outlines organizations that use errors or lapses as an opportunity to learn how to prevent future errors and to foster continuous quality improvement rather than punishing individuals [46,47]. Veterans in our sample described similar experiences (e.g., being given the wrong medication) and the admission of error as courageous. 

Veterans also described a desire for a change in how the system and individual providers view monetary and healthcare benefits administered by VA. Most of what is known about the veterans benefits process, specifically service connection, is found in the posttraumatic stress disorder (PTSD) and the legal literature. Most veterans view the service connection process negatively [48] and research suggests that women, in particular women of color, experience differential outcomes [49,50]. What is not explored is how the nature of service connection and healthcare benefits as a public service influence the way in which providers interact with veterans. Means-tested programs in particular carry stigma whereby recipients expect to be treated negatively [51]. While veterans’ help-seeking carries a different type of stigma (e.g., the stigma of mental health treatment or perceived weakness) [52], the literature has yet to explore the stigma of veterans’ benefits as a public, and sometimes a means-tested [27], program. Such an inquiry might help inform improvement in veterans’ experiences with the service connection process and VA healthcare. Similarly, the fidelity rule establishes that providers must adhere to the principles of the healing relationship [53]. At the macro level, veterans also view the service connection process as keeping the promise made when they enlisted in the military.

The theme of being proactive also emerged from our interviews with veterans exposed to airborne hazards. A more reactive approach to health has been closely identified with a disease-focused model [54]. The VA has placed tremendous effort into shifting away from a disease model to a model of care that works toward physical, mental, and social health goals that are veteran-centered. This Whole Health model includes wellness programs and self-management coaching [55]. The successful implementation of the Whole Health model is reliant upon several factors, including a strong rapport between the veteran and the provider as well as facility-level leadership dedicated to the model [56]. This suggests that VA is already implementing a climate of care that is more proactive, and that support is needed at the facility level (i.e., leadership) in order to successfully move to this model.

Finally, veterans in our sample discussed the importance of advocacy in organizations in terms of both self-advocacy and speaking up for others. The existing literature discusses fear on the part of healthcare providers to engage in advocacy, as well as the investment of time which advocacy requires. Advocacy often involves interactions with management or systems of power, which can result in adverse consequences for providers, including retaliation or dismissal [57]. There is also a fear that nothing will happen as a result. In a study in the United Kingdom, 45% of nurses said that they had spoken with management about patient care concerns but that no changes were made by management [58]. Furthermore, advocacy with colleagues must often take on a delicate balance of obtaining needed resources for one patient while not impairing a collegial relationship required for future patients. Advocacy at both the patient-level and systems-level requires time, a precious commodity for many healthcare providers. In their work with midwives in the National Health Service in the United Kingdom, Finlay et al. [59] noted a challenge balancing the system’s need to efficiently see more clients and client-centered care: less time spent with patients was directly correlated with an inability to provide patient-level advocacy. Collective advocacy with the goal of system-wide change requires the time to network and foster a community. In the case of the Australian healthcare system, physicians advocated for improved access to adult psychiatry beds through data analyses, policy papers, peer reviewed publications, non-profit or professional associations (e.g., the College of Emergency Physicians), and meetings with management, including the Minister of Health [60]. 

Our study included several limitations. Our goal was to improve care for a specific group of veterans who seek care in our clinic for a specific exposure concern (i.e., airborne hazards). Therefore, this quality improvement endeavor should not be construed as generalizable to all veterans, including all veterans with exposure concerns. We approached a small group of veterans to allow for resources to be dedicated to member-checking; additional themes may have emerged from a larger group of respondents.

## 5. Conclusions

Institutional betrayal occurs through wrongdoing, systematic discrimination, actions of law or healthcare agencies or officials, punitive actions against whistleblowers, and a neglect of workplace hazards [1,2,3,4,5]. Military veterans, especially those using VA healthcare, may be particularly susceptible to institutional betrayal and its harm due to the potential for strong identification with or reliance upon this system (e.g., through healthcare, housing, career development, etc.). As discussed, veterans have expressed feelings of betrayal regarding military environmental exposures, related benefits, and their subsequent health services. The Sergeant First Class Heath Robinson Honoring our Promise to Address Comprehensive Toxics Act of 2022 (PACT Act) offers a new opportunity to improve care for veterans with environmental exposure concerns [61]; potentially mitigating further institutional betrayal and providing a transferable example for other sources of betrayal, and further defining and promoting institutional courage. Providers need institutional support to enact institutional courage in their practice. As providers, we can welcome the call to institutional courage described by veterans in this quality improvement project to hold ourselves and others accountable to each other and to veterans, provide proactive outreach to veterans, approach each case uniquely and avoid a one-size-fits all approach to care, value and support advocacy in our care teams, shift conversations around VA healthcare and benefits to educate each other on stigma, and offer safety and support to veterans and colleagues.

## Data Availability

Due to the nature of the interviews, data are not available. Further questions with regard to analysis and methodology can be directed to the first author.
